# Smartphone-Based 3D Indoor Pedestrian Positioning through Multi-Modal Data Fusion

**DOI:** 10.3390/s19204554

**Published:** 2019-10-19

**Authors:** Hongyu Zhao, Wanli Cheng, Ning Yang, Sen Qiu, Zhelong Wang, Jianjun Wang

**Affiliations:** 1 A Key Laboratory of Intelligent Control and Optimization for Industrial Equipment of Ministry of Education, Dalian University of Technology, Dalian 116024, China; 2 School of Control Science and Engineering, Dalian University of Technology, Dalian 116024, China; 3 Beijing Institute of Spacecraft System Engineering, Beijing 100094, China; wjjxy1998@163.com

**Keywords:** pedestrian dead reckoning (PDR), indoor localization, pedestrian navigation, barometer, map matching, particle filter, gait analysis, inertial measurement unit (IMU), inertial sensor, inertial navigation system (INS)

## Abstract

Combining research areas of biomechanics and pedestrian dead reckoning (PDR) provides a very promising way for pedestrian positioning in environments where Global Positioning System (GPS) signals are degraded or unavailable. In recent years, the PDR systems based on a smartphone’s built-in inertial sensors have attracted much attention in such environments. However, smartphone-based PDR systems are facing various challenges, especially the heading drift, which leads to the phenomenon of estimated walking path passing through walls. In this paper, the 2D PDR system is implemented by using a pocket-worn smartphone, and then enhanced by introducing a map-matching algorithm that employs a particle filter to prevent the wall-crossing problem. In addition, to extend the PDR system for 3D applications, the smartphone’s built-in barometer is used to measure the pressure variation associated to the pedestrian’s vertical displacement. Experimental results show that the map-matching algorithm based on a particle filter can effectively solve the wall-crossing problem and improve the accuracy of indoor PDR. By fusing the barometer readings, the vertical displacement can be calculated to derive the floor transition information. Despite the inherent sensor noises and complex pedestrian movements, smartphone-based 3D pedestrian positioning systems have considerable potential for indoor location-based services (LBS).

## 1. Introduction

Nowadays, almost everyone has their own smartphone, as people’s daily life cannot be separated from the convenience provided by some functions of smartphones [1]. Most smartphones have a wealth of sensors or systems built into them, such as the Global Positioning System (GPS), Wi-Fi, photoreceptor, microphone, magnetometer, barometer, inertial sensors (i.e., gyroscopes and accelerometers), etc. Therefore, the potential for using smartphones as portable sensing devices remains to be exploited. Localization and navigation via a global navigation satellite system (GNSS) has become an important part in smartphone´s daily use. However, because of the signal blockage and reflection, although GNSS can provide accurate location information in outdoor environments, there are considerable challenges when it is used in indoor or indoor-like environments, such as inside buildings, tunnels or caves, as well as in urban canyons, deep canopies or dense forests [2]. As people spend most of their time indoors (e.g., at work and at home), it is important to find an infrastructure-free pedestrian positioning solution for indoor location-based services (LBS).

As is known, an inertial positioning system constructed of wearable inertial sensors is a self-contained technique that can track a person’s location in any environments and requires no external infrastructure or additional environmental information. Since almost everyone has a smartphone, it is preferable to use the smartphone’s built-in inertial sensors for indoor pedestrian positioning, rather than the expensive dedicated inertial sensors. As a ubiquitous portable electronic device, a smartphone not only provides a cost-effective platform for pedestrian positioning, but also makes the indoor LBS applications easier to popularize, as people are quite used to using a smartphone for outdoor localization and navigation [3]. With the rapid development of smartphone technology and the increasing number of smartphone users, the smartphone-based positioning system has proved to be valuable in many applications. In the mobile application market, more and more research attention has been paid to an indoor LBS based on smart mobile devices.

Generally, there are two types of inertial positioning techniques, i.e., pedestrian dead reckoning (PDR) and the strapdown inertial navigation system (SINS). However, as the smartphone’s inertial sensors are fabricated using micro-electro-mechanical system (MEMS) technology, the inertial data are polluted by various noises [4], making that the estimates of both PDR and SINS drift significantly over time. For the SINS algorithm, the position is estimated by numerical integration of the accelerometer and gyroscope data at regular time intervals, and the error growth is proportional to the cube of operation time. The strict accuracy requirements in such algorithms justify the cost and size of high-quality inertial sensors. By contrast, as an alternate positioning solution, the PDR algorithm is a cost-effective choice and supposed to better suit the pedestrian’s gait patterns. Given that a foot takes one stride at a time, the PDR mechanization can restrict error growth by propagating position estimates in a stride-wise manner that accords with the walking cadence, rather than over a fixed time interval that corresponds to the sensor sampling frequency. In this way, the error growth is proportional to the number of strides taken.

Relatively speaking, when using the same noisy inertial sensors, PDR features a lower error rate over time than the pure SINS. In some research, foot-mounted SINS aided by the well-established zero velocity updates (ZUPT) technique is implemented to bound the error growth. According to human gait biomechanics, during most types of legged locomotion (such as walking, running, and ascending or descending stairs), each gait cycle has a stance phase, during which the foot is entirely on the ground and its velocity should be zero in ideal conditions [5]. This specific zero-velocity information can be effectively utilized by the ZUPT technique [6,7]. A general framework for context-aided inertial navigation is presented in [8], which exploits the available sources of information in a principled manner. However, the advantages of PDR over SINS are the flexible sensor placement and low requirement of sensor accuracy, which are especially important when using the MEMS inertial measurement units (IMUs) embedded in smartphones [9]. Therefore, PDR has become one of the popular techniques for smartphone-based positioning.

## 2. Related Work

**Figure 1:**
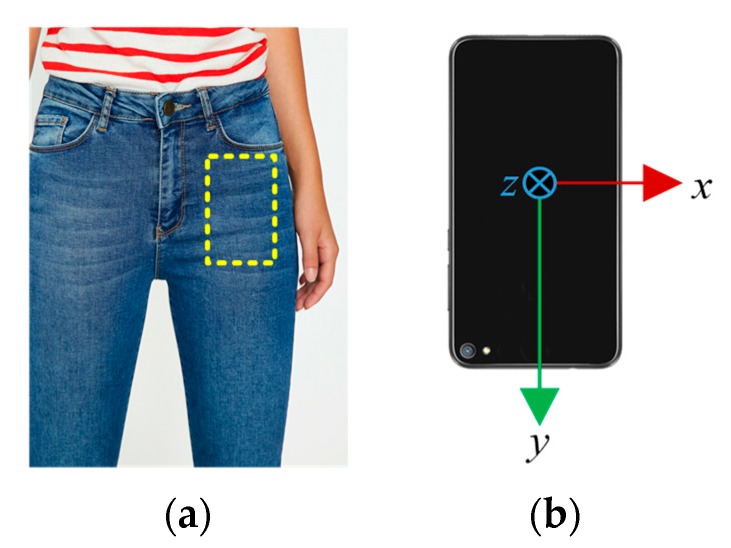
Smartphone’s placement and orientation. (**a**) Common location for carrying a smartphone; (**b**) initial orientation of the smartphone.

Recently, many research works have focused on pedestrian positioning in indoor environments. Some scholars have studied the PDR technology based on handheld smartphones [10]. In our former work [11], we put a smartphone in the front pocket of pedestrians’ pants, since it is a common way for people to carry their smartphones, and then the PDR system is realized based on the sensors embedded in the smartphone, as shown in Figure 1. We aimed to implement a novel PDR system for indoor pedestrian tracking, by using the gyroscope, accelerometer and magnetometer that are embedded in a standard and commercial smartphone. The feasibility and effectiveness of the PDR system are verified with extensive experiments. However, there are two main disadvantages in a PDR system: (1) the positioning result experiences an ever-increasing error, especially the heading drift that can accumulate several degrees of orientation error after even one minute; (2) it only estimates the position in 2D space and cannot locate the pedestrian’s position in 3D space. To overcome these limitations, multi-modal data fusion techniques have usually been utilized, by fusing different sensors that are embedded in smartphones and various aiding information that are available to users, which is a promising way to improve the indoor-positioning performance.

For smartphone-based PDR, due to the error characteristics of the smartphone’s built-in sensors, there exists a considerable amount of heading error caused by gyroscope bias. Many sensor fusion algorithms have been utilized to bound the heading error, such as the extended Kalman filter (EKF) [12], complementary filter (CF) [13], and gradient descent algorithm (GDA) [14,15], by fusing gyroscopes with accelerometers and magnetometers. Map information has also been widely used to improve the PDR results via map-matching algorithms [16], especially the global wall orientation data [17,18]. The impact of map awareness on localization performance has been investigated in [19] from a theoretical perspective, evaluating different accuracy bounds. When the walking trajectory is estimated and matched onto the floor plan of a building, some part of the estimated trajectory may cross through the walls, which is obviously not in line with the actual walking behavior. For this reason, a particle filter-based map-matching algorithm is often used to solve the wall-crossing problem in indoor buildings [20,21,22,23].

However, most of the aforementioned research focuses on 2D positioning. Inspired by the height estimation of unmanned aerial vehicles (UAV) based on barometers [24], some studies showed that the pedestrian height could also be obtained by using a barometer [25,26]. It is believed that the 2D PDR can be easily extended for 3D applications, without major change of software and alteration of hardware. The smartphone’s built-in barometer can achieve sub-meter resolution [27], which is not small enough to capture the height change during a single gait cycle, but sufficient for floor transition detection. As expected, the accuracy of the barometer heights clearly outperforms that of the GPS heights, especially in indoor environments. Unlike the accelerometer-based altimetry, the barometer reading is an absolute value measured from sea level, which has the advantage of not accumulating errors over time. Unlike other absolute altimetry techniques, the barometers require no external infrastructure, such as GPS satellites, visual patterns, or acoustic beacons. Unlike the on-board barometer installed in a UAV, the smartphone’s built-in barometer has several unique advantages: (1) as the phone is placed inside the user’s pocket close to human body, the temperature drift is no longer an issue; (2) as the phone will not be exposed to outdoor air, the barometer is less affected by the airflow; (3) as there is a limited range of pedestrian movement in vertical direction, some simplifications can be made during the pressure-height conversion. 

This paper is a continuation of our previous study [11], which focused on the implementation of a 2D PDR based on a pocket-worn smartphone, by using the inertial sensors and magnetometer embedded in the smartphone. This study aims to improve the PDR system in two aspects: (1) introduce a particle filter-based map-matching algorithm by fusing the indoor map data, which is supposed to solve the wall-crossing problem and thereby improve pedestrian positioning accuracy; (2) extend the smartphone-based pedestrian tracking applications from 2D to 3D space in indoor environments by fusing the smartphone’s built-in barometer, which can be used to track the pedestrian’s vertical displacement and, therefore, obtain the height information for floor transition prediction and detection. A large number of experiments have been carried out, the results of which verify the feasibility and effectiveness of the particle filter-based map-matching algorithm for horizontal trajectory refinement, as well as the barometer-based height estimation algorithm for vertical trajectory determination. 

## 3. Coordinate Systems and Initial Alignment

This section gives a brief description of the involved coordinate systems and the system initial alignment. The sensor error calibration was detailed in our previous study [11], which is not described here for the sake of brevity.

### 3.1. Coordinate Systems

**Figure 2:**
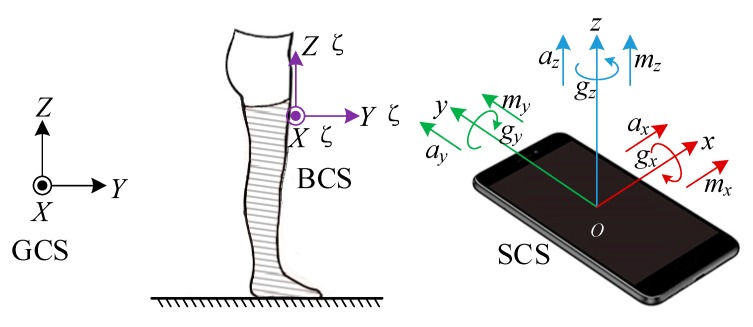
Illustration of the global, body, and sensor coordinate systems.

As shown in Figure 2, in this implemented PDR system, three coordinate systems are established, i.e., a global coordinate system (GCS), a body coordinate system (BCS), and a sensor coordinate system (SCS). These three coordinate systems are defined in the following. We measured all the inertial data in SCS and calculated all the PDR states in the GCS.

(1) The GCS, known as east-north-up (ENU) coordinate system, is fixed to the Earth’s surface with its three axes pointing to the directions of east, north, and up respectively and denoted by XYZ;

(2) The BCS is fixed to the user’s body segment with its three axes pointing to right, forward, and upward directions respectively, which is denoted by $X^{\prime }Y^{\prime }Z^{\prime }$;

(3) The SCS is fixed to the pocket-worn smartphone with its three axes parallel to the built-in sensor’s axes, which is denoted by xyz.

In the initial state, the BCS can be aligned with the GCS. However, there are no strict restrictions on the location and direction in which the pedestrian can place the phone in his/her pants’ pocket. Therefore, the three axes of the SCS are probably not parallel to that of the GCS, and the heading of smartphone is usually not aligned with the true direction of walking. As shown in Figure 1 and Figure 2, the smartphone is kept in the pedestrian’s front pocket vertically, with the positive direction of y-axis downward and the positive direction of z-axis backward. Thus, the SCS has an initial roll angle of approximately 180 degrees about the Y-axis of the GCS, and an initial pitch angle of approximately −90 degrees about the X-axis of the GCS. 

### 3.2. Initial Alignment

As PDR mechanization is a relative positioning scheme, its performance is greatly dependent on the accuracy of system initialization. In our study, an initial alignment method based on multi-sensor fusion is proposed. As shown in Figure 2, during initial alignment, the pedestrian is instructed to face north and stand still for a while (e.g., a few seconds). Then, the initial Euler angles can be obtained as

(1) $$\left\{ \begin{array}{l} \varphi =\arctan\;2(a_{y},a_{z})\\ \theta =\arctan\;2(a_{x},-a_{y}s\phi -a_{z}c\phi )\\ \psi =\arctan\;2(m_{x}c\theta +m_{y}s\theta s\phi +m_{z}s\theta c\phi ,m_{y}c\phi -m_{z}s\phi ) \end{array}\right.,$$

where φ, θ, and ψ are the roll, pitch, and yaw angles, respectively; s and c are the sine and cosine functions, respectively; $a_{x}$, $a_{y}$, and $a_{z}$ are the average value of the specific force measured by the accelerometer, respectively; $m_{x}$, $m_{y}$ and $m_{z}$ are the average value of the magnetic field density measured by the magnetometer, respectively.

It is known that the attitude of an object can be equivalently represented by Euler angles, rotation matrix, and quaternion. In our study, considering the so-called gimbal lock problem, the quaternion is used to describe the attitude, which can be represented in hypercomplex form as

(2) $$q=q_{0}+q_{1}i+q_{2}j+q_{3}k,$$

where $q_{0}$, $q_{1}$, $q_{2}$, and $q_{3}$ are real numbers, and i, j, and k are complex operators.

As three coordinate systems are involved, three types of quaternions are introduced accordingly to describe the relative orientations between them, which are 

(1) ${}_{B}^{G}q$ describes the rotation between the BCS and GCS;

(2) ${}_{S}^{G}q$ describes the rotation between the SCS and GCS;

(3) ${}_{S}^{B}q$ describes the rotation between the SCS and BCS.

As is mentioned above, as the BCS and GCS are oriented nearly parallel to each other in the initial state, there is

(3) $${}_{B}^{G}q_{init}\approx [1\;0\;0\;0].$$

Besides, as the smartphone is placed in a substantially fixed position with respect to the pedestrian’s thigh, ${}_{S}^{B}q$ is assumed to remain unchanged during the walking trial and can be expressed as

(4) $${}_{S}^{B}q\approx {}_{B}^{G}q_{init}⊗{}_{S}^{B}q={}_{S}^{G}q_{init}.$$

Then, the concerned quaternion ${}_{B}^{G}q$ that describes the thigh rotation from the BCS to GCS during walking can be obtained by

(5) $${}_{B}^{G}q={}_{S}^{G}q⊗{}_{B}^{S}q={}_{S}^{G}q⊗{\left({}_{S}^{B}q\right)}^{*}={}_{S}^{G}q⊗{\left({}_{S}^{G}q_{init}\right)}^{*},$$

where ${\left(\cdot \right)}^{*}$ denotes the quaternion conjugate operation.

## 4. Pedestrian Positioning Implementation

**Figure 3:**
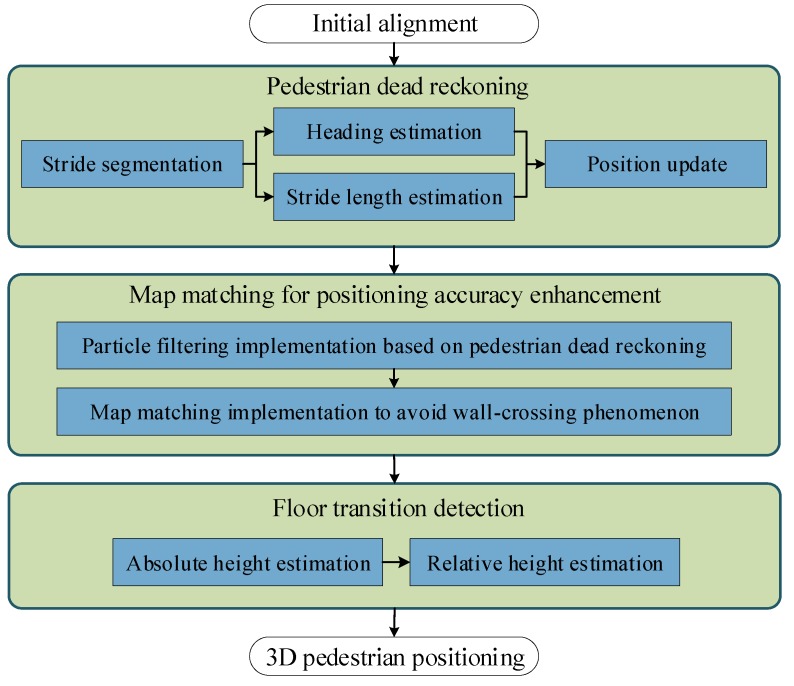
Block diagram of the proposed 3D pedestrian positioning algorithm.

In this section, the PDR system is implemented first to track pedestrian’s 2D position, then the map information is used to refine tracking results, and finally the height information is introduced to realize 3D positioning. The block diagram of the proposed algorithm is shown in Figure 3.

### 4.1. Pedestrian Dead Reckoning (PDR)

PDR is a relative positioning technique, which propagates a position based on the estimated walking direction and distance over each stride on a 2D plane. There are three important tasks during PDR, i.e., stride detection, stride length estimation, and heading determination, as detailed below.

#### 4.1.1. Attitude Estimation

The attitude angles can be obtained by integrating the gyroscope measurement over time, by using a quaternion-based attitude estimation algorithm. Whenever the quaternion is updated by using the latest gyroscope measurement, the attitude angles can be calculated by

(6) $$\{\begin{array}{l} \begin{array}{l} \varphi =\arctan\;2\;(2(q_{0}q_{1}+q_{2}q_{3}),1-2(q_{1}^{2}+q_{2}^{2}))\\ \theta =\arcsin\left(2\left(q_{0}q_{2}-q_{1}q_{3}\right)\right) \end{array}\\ \psi =\arctan\;2\;(2(q_{0}q_{3}+q_{1}q_{2}),1-2(q_{2}^{2}+q_{3}^{2}))-D \end{array},$$

where D is the Earth’s magnetic declination.

In our previous study, to bound the error growth of attitude estimation, an improved sensor fusion algorithm is proposed based on a gradient descent method. The original quaternion-based GDA is presented by Madgwick et al. [14], which allows the measurements of accelerometer and magnetometer to be used in an analytically derived and optimized gradient descent algorithm to compute the direction of the gyroscope measurement error as a quaternion derivative. However, there is very little discussion of the assumption made in GDA that the magnetometer measures only the Earth’s magnetic field. However, the measurements of magnetometer can be easily distorted by environmental magnetic interference (e.g., electronic apparatus), making the magnitude of magnetometer measurements fluctuate around the Earth’s magnetic field strength. In practice, some fluctuations are too large to be technically utilized.

To deal with the magnetic fluctuations, a threshold-based strategy is implemented to distinguish the usable and unusable magnetometer measurements. At each time instant, a usable magnetometer measurement can be declared if

(7) $$\left|\left\|m_{t}\right\|-m_{Earth}\right|\leq lim_{mag},$$

where t is the time instant, $m_{t}$ is the magnetic field strength measured by the magnetometer, and $\left\|m_{t}\right\|$ is the 2-norm of $m_{t}$; $m_{Earth}$ is the strength of the earth’s magnetic field [28]; $lim_{mag}$ is the predefined threshold to determine if the magnetometer measurement is usable.

**Figure 4:**
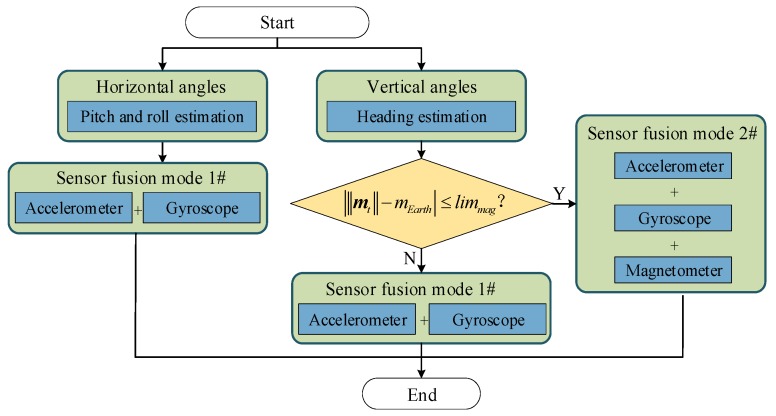
Attitude estimation algorithm by considering the severity of magnetic interference.

Thus, the original GDA can be improved by considering the magnetic interference on heading estimation, which switches among different types of sensor fusion strategies, as shown in Figure 4. 

#### 4.1.2. Stride Segmentation

**Figure 5:**
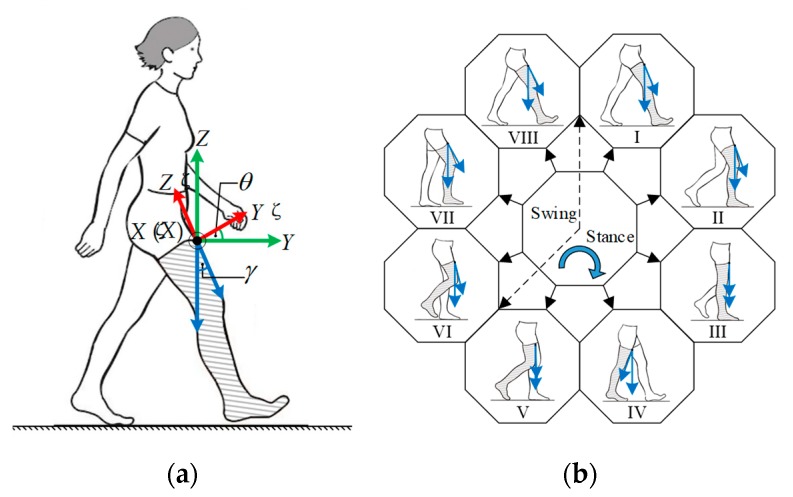
Thigh angle changes during a stride cycle. (**a**) Thigh angle definition; (**b**) key events and phases of a gait cycle.

Human walking is a complex kinematic and dynamic activity, as human structure features a high degree of freedom (DOF) and 3D deformable frames, and the human gait is multifactorial in terms of its control mechanisms governed by the neuromuscular system. Normally, gait has a periodic and regular pattern. A gait cycle can be defined as the period between two successive identical events of the same foot, which can be termed as a stride and consists of two steps [29]. Each gait cycle has a series of ordered gait events and associated gait phases, which are referred to as temporal gait parameters as they occur at specific temporal locations. According to the specific purpose, a whole gait cycle can be divided into several phases by gait events, from two to eight or even more [30]. An example of dividing a gait cycle into eight phases is shown in Figure 5, which are initial contact (I), loading response (II), mid-stance (III), terminal stance (IV), pre-swing (V), initial swing (VI), mid-swing (VII), and late swing (VIII). Terminologically, any event could be specified as the start of a gait cycle, as various gait events follow each other continuously and smoothly in a specific order.

Based on the knowledge of human gait cycle, body-worn sensors can be used to detect the occurrence of strides, and provide a means to estimate the distance and direction in which the stride is taken. As gait motion mainly occurs in the sagittal plane, it can be observed that the pitch angle θ has more prominent features than the other two attitude angles for gait event indication. As illustrated in Figure 5, a new angle that is denoted by γ and named as thigh angle can be introduced for gait detection, which describes the thigh swings away from vertically downward direction. By contrast, although there is θ=γ, the angle γ is more in line with people’s habit of understanding the thigh rotation in the sagittal plane.

For PDR implementation, the concern is not the detailed gait phases or subphases, but only one kind of gait event that can effectively realize the segmentation of successive strides. The key events when the thigh angle reaches its positive peaks have been detected for this purpose, which also serve as the onsets of gait cycles. Thus, the pedestrian’s location can be updated periodically when the positive peaks are identified.

#### 4.1.3. Stride Length Estimation

**Figure 6:**
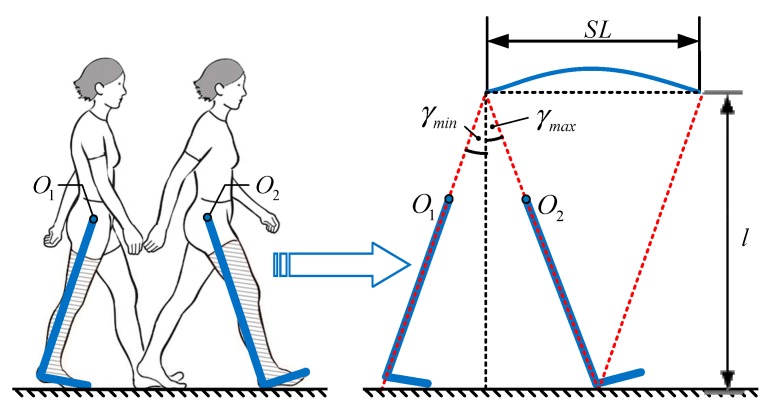
Gait movement during a stride and associated gait model.

Stride length estimation is a critical step of PDR mechanization. Discrete integration of gravity-compensated acceleration introduces inevitable drift as a function of time, due to the bias and random noise inherent in the smartphone’s built-in sensors. For estimating stride length using inertial sensors, human gait is usually modeled as a pendulum [31]. In this paper, human gait is modeled by an inverted pendulum of a kneeless biped on the sagittal plane, as shown in Figure 6. In this model, $O_{1}$ and $O_{2}$ are the body’s center of mass (COM) at the start and end events of a stride, respectively. Accordingly, $\gamma_{max}$ and $\gamma_{min}$ are the thigh angles at these two events, which corresponds to the maximum (positive peak) and minimum (negative peak) thigh angles during the stride, respectively.

Thus, the stride length can be estimated by

(8) SL=l⋅f(γ)+b,

where *SL* is the stride length, l and b are two model parameters that are needed to be identified, and $f(\gamma )=\tan(\gamma_{max})+\tan(-\gamma_{min})$.

#### 4.1.4. Position Update

**Figure 7:**
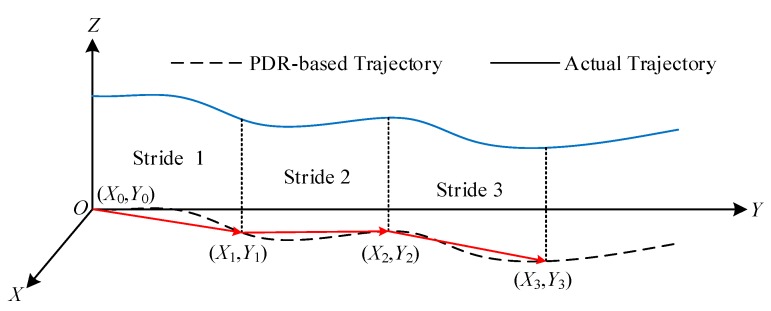
Schematic diagram of pedestrian dead reckoning.

When the three tasks, i.e., the estimation of attitude angles, the segmentation of successive strides, and the calculation of stride length, have been accomplished in a satisfactory manner, the PDR can be implemented successfully, as illustrated in Figure 7.

Thus, with known position at the end of the previous stride, as well as the estimates of stride length and heading angle of the current stride, the latest position can be updated by

(9) $$P_{k}^{2D}=\left[\begin{array}{c} P_{k}^{X}\\ P_{k}^{Y} \end{array}\right]=\underset{P_{k-1}}{\underset{︸}{\left[\begin{array}{c} P_{k-1}^{X}\\ P_{k-1}^{Y} \end{array}\right]}}+\underset{\Delta P_{k}}{\underset{︸}{\left[\begin{array}{c} SL_{k}\cos(\psi_{k})\\ SL_{k}\sin(\psi_{k}) \end{array}\right]}},$$

where k is the number of stride taken, $P_{k}^{2D}$ is the position at the end of the current stride in 2D space, $P_{k-1}$ is the position at the end of the previous stride, $\Delta P_{k}$ is the displacement over the current stride; $P_{k}^{X}$ and $P_{k}^{Y}$ represent the displacements along east and north directions of GCS with respect to that start position, respectively.

### 4.2. Map Matching for Positioning Accuracy Enhancement

In this subsection, the original PDR system is enhanced by introducing a particle filter-based map-matching algorithm to solve the problem of estimated pedestrian’s path passing through the walls of a building.

#### 4.2.1. Particle-Filtering Implementation

As a relative positioning technology, the PDR error will accumulate over time during walking. In outdoor environments, the requirement for positioning accuracy is often not strict, where a positioning error of a few meters will not cause too much problem in user experience. However, in indoor environments, the building structure is relatively compact and the positioning target is relatively dense, and therefore a small positioning error might cause poor user experience. When mapping the localization results on the map, there might be problems, such as wall-crossing, which is impossible for human beings. 

This paper aims to refine the positioning results by particle filtering, which is a filtering method based on the Monte Carlo method and recursive Bayesian estimation. Particle filter has been applied in different applications, such as video tracking and localization [32]. The core idea of the particle filter is to estimate the conditional probability density using the Monte Carlo simulations and the importance sampling techniques. In this study, maps are used to reject the incorrect particles that give rise to the wall-crossing trajectories during particle resampling. According to the PDR mechanization, the state transition model of the particle filter can be derived as

(10) $$\left[\begin{array}{c} {}^{\left(i\right)}P_{k}^{X}\\ {}^{\left(i\right)}P_{k}^{Y}\\ {}^{\left(i\right)}\psi_{k} \end{array}\right]=\left[\begin{array}{c} {}^{\left(i\right)}P_{k-1}^{X}\\ {}^{\left(i\right)}P_{k-1}^{Y}\\ {}^{\left(i\right)}\psi_{k-1} \end{array}\right]+\left[\begin{array}{c} {}^{\left(i\right)}{SL}_{k}\cos({}^{\left(i\right)}\psi_{k})\\ {}^{\left(i\right)}{SL}_{k}\cos({}^{\left(i\right)}\psi_{k})\\ \Delta {}^{\left(i\right)}\psi_{k} \end{array}\right],\;$$

where superscript (i) indicates the i-th particle, $\Delta \psi_{k}$ is the heading change over the k-th stride.

In Equation (10), $\left[{}^{\left(i\right)}P_{k}^{X},\;{}^{\left(i\right)}P_{k}^{Y}\right]$ is the state vector, where i=1,2,…,N and N is the number of particles; $\left[SL_{k},\;\Delta \psi_{k}\right]$ is the input vector obtained in the PDR process. Thus, the measurement model of particle filter can be expressed as

(11) $$\left[\begin{array}{c} S{\hat{L}}_{k}\\ \Delta {\hat{\psi }}_{k} \end{array}\right]=\left[\begin{array}{c} SL_{k}\\ \Delta \psi_{k} \end{array}\right]+\left[\begin{array}{c} \sigma_{SL}^{2}\\ \sigma_{\Delta \psi }^{2} \end{array}\right],$$

where $\sigma_{SL}^{2}$ and $\sigma_{\Delta \psi }^{2}$ are the noises of stride length and heading change estimates.

At this point, the state transition model and measurement model of the particle filter have been constructed based on the PDR results.

#### 4.2.2. Map-Matching Implementation

**Figure 8:**
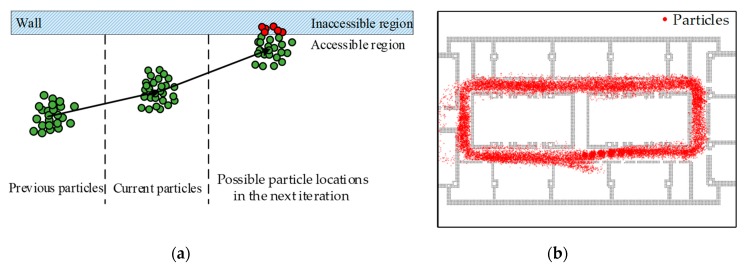
Schematic diagram of particle filtering. (**a**) Phenomenon of particles passing through a wall; (**b**) distribution of particle clouds during particle filtering along a closed rectangular path.

If indoor positioning technology is to be applied in practice, an indoor map is the basic and indispensable information. Otherwise, the positioning results can only be abstracted as points in a coordinate system, but cannot be transformed into geographic information with practical significance. In indoor environments, only the combination of the positioning results and the specific location of a room or a corridor can reflect the technical value of indoor positioning. Therefore, this paper considers the use of indoor map-matching technology to improve positioning performance. Map-matching technology, also referred to as map-aiding technology in some literature, is a pseudo-measurement technology, which can make position correction using software. As demonstrated in Figure 8, during particle filtering, when the particles propagate to the next time step, some particles (filled with red) might pass through the walls.

It is an undisputed fact that pedestrian cannot walk through a wall or reach an inaccessible area. The particles that reach the unreachable area should be regarded as invalid particles, and their weight should be set to zero. Thus, the particle weight is updated with the following rule

(12) $$\omega_{k}^{\left(i\right)}=\left\{ \begin{array}{l} 0,\;\mathrm{Inaccesible}\;\mathrm{region}\\ \omega_{k}^{\left(i\right)},\;\mathrm{Accesible}\;\mathrm{region} \end{array}\right.,$$

where $\omega_{k}^{\left(i\right)}$ is the particle weight.

In summary, the particle filter-based map-matching algorithm is supposed to eliminate the non-matching particles, and thereby to solve the wall-crossing problem, which is quite effective in indoor environments that have lots of walls or inaccessible areas.

### 4.3. Floor Transition Detection

The smartphone’s built-in barometer provides a strong support of the PDR solution with height information. Obtaining a pedestrian’s height information from measured atmospheric pressure is a complex and challenging process. A fundamental question for the use of the barometer in indoor environments is its suitability for floor identification [33]. A barometric height equation is presented in [34], which can convert the measured air pressure into the height information, as given by

(13) $$h_{k}=\left(1-{\left(H_{k}/H_{\mathrm{ref}}\right)}^{\frac{R\cdot S}{D\cdot G}}\right)\cdot \frac{T_{\mathrm{ref}}}{S},$$

where $h_{k}$ is the relative height with respect to sea level; $H_{k}$ is the measured atmospheric pressure, $H_{\mathrm{ref}}$ is the reference pressure of sea level; R is the universal gas constant, S is the temperature decrement rate, D is the molar mass of dry air, G is the local gravity acceleration, and $T_{\mathrm{ref}}$ is the standard temperature at sea level.

**Figure 9:**
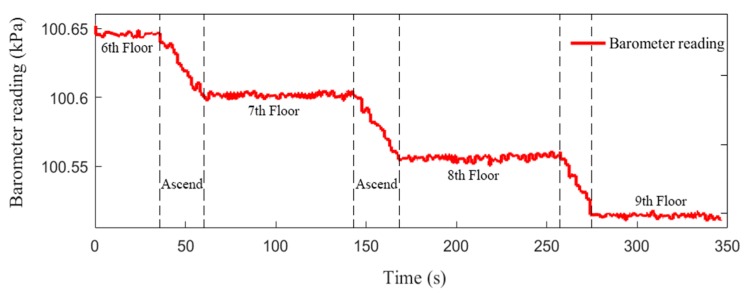
Floor detection with barometer measurement on the way upstairs.

Since the parameters involved in Equation (13) are easily affected by the characteristics of meteorological environment, the absolute sea level height obtained might be not accurate enough. However, the barometer is accurate enough in measuring small pressure differences. In practical applications, some assumptions are usually made to the atmospheric parameters, e.g., an average standard atmospheric pressure at sea level (i.e., 1013.25 kPa) is used and the pressure is assumed to depend only on the current height. Field test has been conducted along a 3D path including six flights of stairs between four floors in a building. The floors were visited with the sequence of 6–7–8–9, and Figure 9 shows the raw barometer measurements on the way upstairs. As is seen in Figure 9, the floors can be readily distinguished from each other, and the measurements fluctuate within a small range on each floor. As the barometer sensor is less affected by frequent minor movements of a body-attached smartphone, these fluctuations are mainly caused by the sensor’s limited accuracy.

For 3D tracking, the measured atmospheric pressure in initial stage is used as a reference for conversion from pressure into height. In this manner, the relative height can be estimated based on the difference between current atmospheric pressure and initial atmospheric pressure, as given by

(14) $$\Delta h_{k}=h_{k}-h_{0},$$

where $h_{0}$ is the pedestrian’s height estimated at initial stage with respect to sea level, and $\Delta h_{k}$ is the relative height with respect to initial level.

Thus, in the 3D indoor positioning system, a pedestrian’s initial height is equal to zero, i.e., $\Delta h_{0}=0$. Based on Equations (13) and (14), the desired pedestrian’s height can be estimated. Finally, the position update equation presented in Equation (9) can be augmented to realize the positioning in 3D space, which is given as

(15) $$P_{k}^{3D}=\left(\begin{array}{c} P_{k}^{X}\\ P_{k}^{Y}\\ P_{k}^{Z} \end{array}\right)=\underset{XY\text{-}\mathrm{plane}}{\underset{︸}{\left(\begin{array}{c} P_{k-1}^{X}\\ P_{k-1}^{Y}\\ 0 \end{array}\right)+\left(\begin{array}{c} SL_{k}\cos(\psi_{k})\\ SL_{k}\sin(\psi_{k})\\ 0 \end{array}\right)}}+\underset{Z\text{-}\mathrm{axis}}{\underset{︸}{\left(\begin{array}{c} 0\\ 0\\ \Delta h_{k} \end{array}\right)}},$$

where $P_{k}^{3D}$ is the 3D position at the end of the k-th stride.

## 5. Experiment and Result

In this section, the experimental setup is firstly described, then the process and results of the experiment are presented, and finally some discussions are made on the experimental results.

### 5.1. Experimental Setup

**Figure 10:**
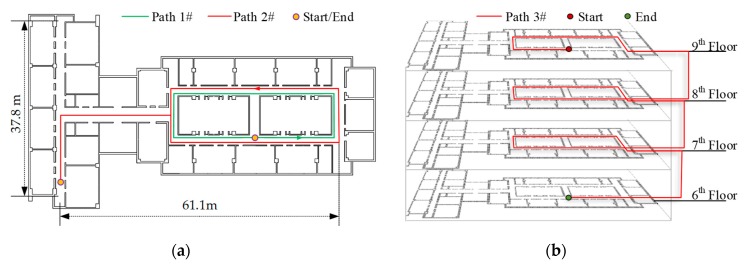
Horizontal floor plan and predefined reference paths. (**a**) 2D path; (**b**) 3D path.

To evaluate the smartphone-based 3D positioning system, experiments were performed in a typical indoor environment of a 16-floor concrete building. The smartphone used for data collection is iPhone X (Apple Inc., Cupertino, CA, USA), which has the dimensions of 143.6 mm × 70.9 mm × 7.7 mm and a weight of 174 g, and which is equipped with all the necessary sensors including three-axis accelerometer, three-axis gyroscope, three-axis magnetometer, and barometer. All experimental measurements were sampled at a frequency of 50 Hz. The measurements was first stored in the smartphone’s memory, and then processed using MATLAB (MathWorks Inc., Natick, MA, USA). Three reference paths were planned, i.e., two 2D paths and one 3D path, as shown in Figure 10.

For paths 1# and 2#, except the start and end points that coincide to form a closed-loop path, a number of reference points are added respectively to provide the ground truth points at which the position errors can be determined for algorithm evaluation. When passing through the reference points, the subjects were asked to step over them as strictly as possible. For path 3#, the floors were visited with the sequence of 6–7–8–9, and the start and end points coincide in the vertical direction to form a closed-loop path in the plan view.

### 5.2. Experimental Results

In this subsection, three types of test are presented, to evaluate the above-discussed algorithms for pedestrian dead reckoning, map matching, and floor transition detection, respectively.

#### 5.2.1. Pedestrian Dead Reckoning Test

**Figure 11:**
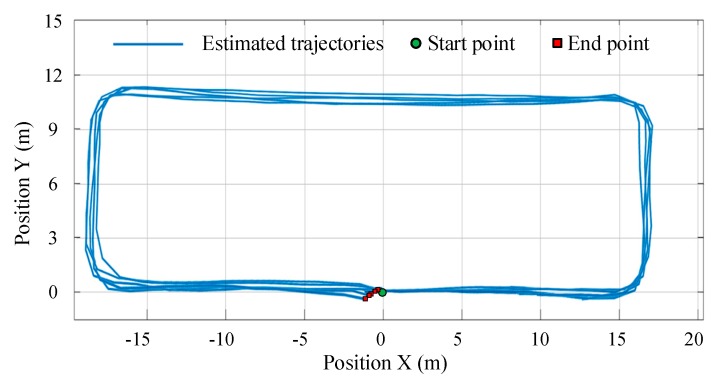
Estimated trajectories of all trials along path 1#.

To collect abundant experimental data, we recruited five experimenters to do this experiment. Each subject was asked to walk through the corridor along path #1 once, which is 92.46 m and has four 90-degree turns. While walking, the pedestrian does not need to turn exactly ±90 degrees, but to turn comfortably and naturally. For convenience of analyzing of the experimental results, the start point is located at the origin of GCS, and the initial heading is along the X-axis of GCS. At the end of each trial, the subject was instructed to return to the start position. Thus, the end point should coincide with the start point at the origin (0, 0). The estimated trajectories along path 1# are shown in Figure 11, which indicate that the smartphone PDR can effectively track the pedestrian’s location in the indoor environment, although with inevitable heading drift.

For closed-loop walking, the positioning performance can be evaluated with the return position error, which is quantified by the difference between the start and end positions of the estimated trajectory, with smaller values indicating less drift. Detailed experimental results are summarized in Table 1. As is shown, the strides of all subjects are correctly detected, which demonstrates the effectiveness of the proposed thigh angle-based stride detection method. The estimated walking distance has a mean value of 90.58 m, which is shorter than the predefined path length, as it is difficult for the pedestrian to turn perfectly along a right-angle corner and thereby the estimated trajectories have no strict 90-degree turn but with a certain turning radius at each corner. The mean absolute return position error is 0.67 ± 0.4 m, while the maximum, minimum, and mean relative return position errors are 1.19%, 0.53%, and 0.77%, respectively.

#### 5.2.2. Map-Matching Algorithm Test

In this experiment, the two 2D paths in an indoor corridor are involved, i.e., path 1# and path 2#. The particle filter-based map-matching is implemented to refine the trajectories that are estimated by the PDR algorithm. For particle filtering, map data need to be imported in advance. The southern boundary of the map area overlaps with the X-axis of the GCS, while the western boundary overlaps with the Y-axis of the GCS. Since deviations from the designated path are unavoidable, some reference points are set between the start and end points to provide more ground truth.

(1) Walking along path 1#

**Figure 12:**
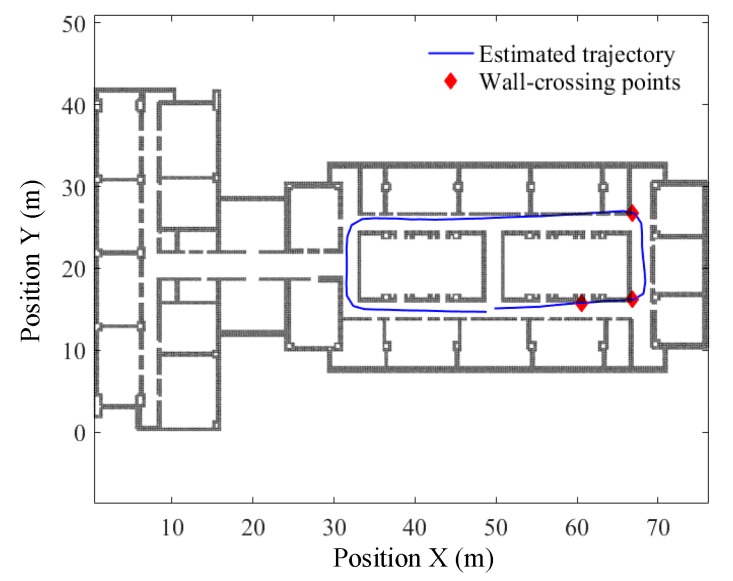
Estimated trajectory with wall-crossing points along path 1#.

Taking the data that yields the worst PDR result (i.e., the walking data of the 3rd subject, as shown in Table 1) for example, to ensure that the start point of the predefined path coincides with its relative location on the map, the start point is shifted to (49.8, 15.1) and, accordingly, the end point is shifted to (48.82, 14.66). The floor map and the estimated walking trajectory are drawn in the same coordinate system, as shown in Figure 12. As can be seen, the estimated trajectory passes through the walls three times, which have been marked with a red rhombus. Figure 12 also demonstrates the accuracy of wall-crossing detection, in which all wall-crossing points in the map are detected. 

**Figure 13:**
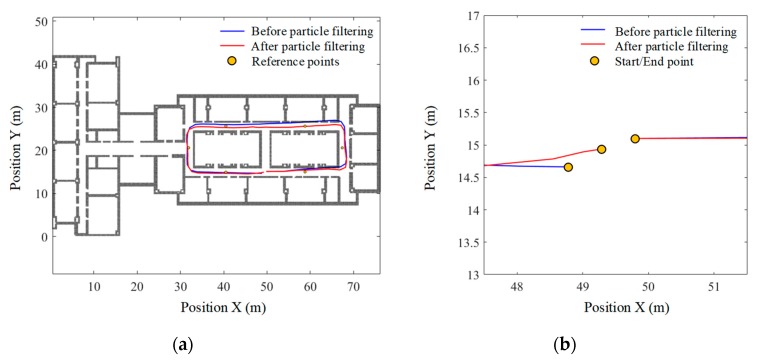
Trajectories estimated before and after particle filtering along path 1#. (**a**) Original view of whole trajectories; (**b**) partial-enlarged view of final positions.

In this test, 500 particles are selected for map matching, and the estimated pedestrian trajectories before and after particle filtering are shown in Figure 13. Detailed examination of Figure 13b shows that the particle filter-based map-matching algorithm reduces the absolute return position error from 1.07 m to 0.34 m, and the relative error from 1.19% to 0.37%. The perpendicular distance errors between the reference points and the estimated trajectories are shown in Table 2, which are calculated before and after particle filtering respectively. As is seen, the mean absolute distance error at the six reference points before particle filtering is 0.61 ± 0.64 m, while the mean relative distance error is 0.67%. After particle filtering, the mean absolute distance error reduces to 0.22 ± 0.51 m, and the mean relative distance error is reduced to 0.24%. These results indicate that particle filtering significantly improves the positioning accuracy over the whole walking process.

(2) Walking along path 2#

**Figure 14:**
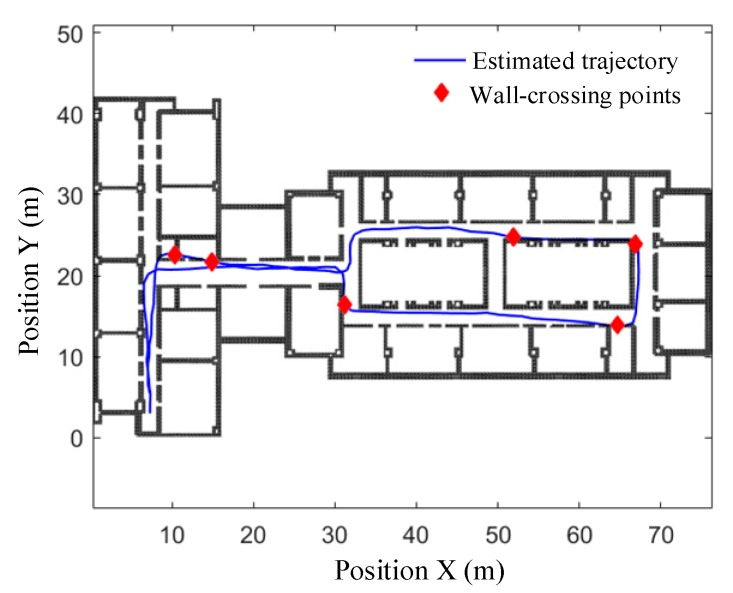
Trajectory with wall-crossing points along path 2#.

**Figure 15:**
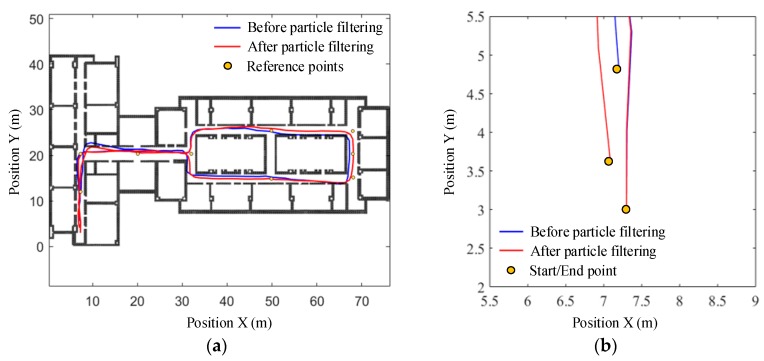
Trajectories estimated before and after particle filtering along path 2#. (**a**) Original view of whole trajectories; (**b**) partial-enlarged view of final positions.

Path 2# is 175.26 m long and includes eight turns, which is more complex than path 1# with longer length and more turns. During walking, subjects not only have to pass through the busiest floor corridor, but also must be close to the power control room, elevator, and other electromagnetic interference environment. The total number of actual strides is 134 and the associated walking distance is 174.63 m (shorter than the predefined path length). The start point is matched to (7.3, 3.0) in the GCS, and the estimated end point is (7.2, 4.8). Similarly, 500 particles are selected for map matching, and the estimated trajectories before and after particle filtering are shown in Figure 14 and Figure 15.

It can be seen in Figure 15b that the particle filter-based map matching reduces the absolute return position error from 1.8 m to 0.67 m, and the relative error from 1.03% to 0.38%. The perpendicular distance errors between the reference points and the estimated trajectories before and after particle filtering are shown in Table 3. It can be seen that the mean absolute distance error at the nine reference points before being filtered is 0.80 ± 0.60 m, while the mean relative distance error is 0.46%. After particle filtering, the mean absolute distance error reduces to 0.18 ± 0.34 m, and the mean relative distance error is reduced to 0.10%. These results once again proves the feasibility and effectiveness of the particle filter-based map-matching algorithm.

#### 5.2.3. 3D Pedestrian Positioning Test

**Figure 16:**
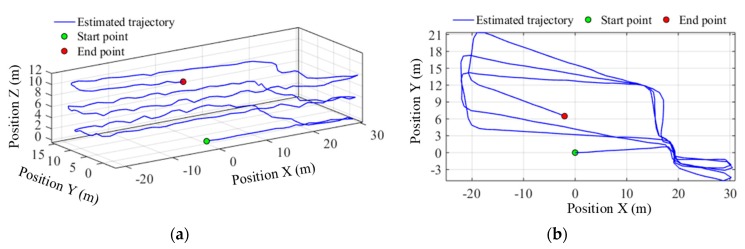
Trajectory estimated before particle filtering along path 3#. (**a**) 3D View; (**b**) plan view.

**Figure 17:**
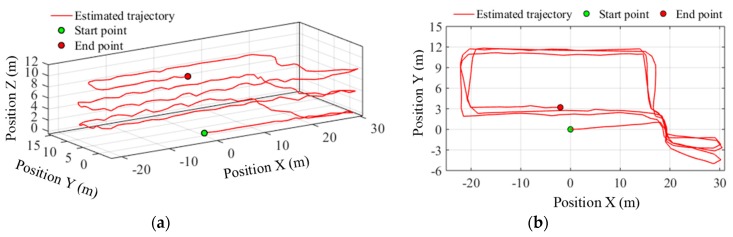
Trajectory estimated after particle filtering along path 3#. (**a**) 3D View; (**b**) plan view.

To verify the performance of the 3D indoor positioning algorithm, there is stair climbing in the predefined path, i.e., path 3#, which can also evaluate the accuracy of the smartphone’s built-in barometer for floor transition detection in indoor environments. The total path includes six flights of stairs between four floors and four sets of horizontal trajectories on each floor. The pedestrian started walking along a straight line in the corridor on the sixth floor to the staircase, and then ascended the stairs from the bottom of the staircase. On the way up, on the seventh and eighth floor, the subject left the staircase, walked along a closed rectangular path (i.e., path 1#) and retraced the route back to the staircase. On the ninth floor, the pedestrian also walked along the rectangular path, but stopped earlier at the point that has the same horizontal coordinates as the start point. The estimated trajectories before and after particle filtering are shown in Figure 16 and Figure 17 respectively. According to the positioning results, the estimated walking distance in 3D space was 363.22 m, and the particle filter-based map matching reduces the absolute final position error from 7.18 m to 4.45 m, and the relative error from 1.98% to 1.22%. More importantly, the heading drift is also effectively suppressed, which is especially noticeable for long duration walking or continual floor transition.

**Figure 18:**
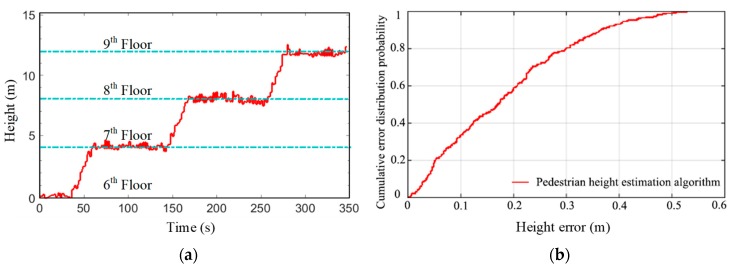
Floor detection results with barometer. (**a**) Estimated walking height on the way upstairs; (**b**) cumulative error distribution curve of height estimation.

During walking, the pedestrian’s height estimates is shown in Figure 18. As the pedestrian starts walking from the sixth floor, the relative height on the sixth floor is zero. In this experimental building, the height between adjacent floors is 4 m. As seen in Figure 18, the height estimation algorithm achieves excellent results for floor transition detection. To further evaluate the height estimation algorithm, the estimated walking heights on the three floors are compared with their reference values. The cumulative error distribution curve of height estimates shows that the algorithm has a 50% probability that the height error is less than 0.17 m, and an 80% probability that the error is less than 0.3 m. This error level will not cause incorrect floor identification.

### 5.3. Discussion

Many scholars have studied PDR based on smartphones and there are many excellent works worthy of attention. However, it is difficult to directly compare the results from different studies, as the system performance depends on many factors, such as system setup, ground surface, and trajectory. Even though, our research has several advantages over those in the state of the art.

(1) Compared with the simple paths in other PDR studies (e.g., in [9,35]), extensive experiments were conducted in our work, with a total walking distance up to 1001.56 m and along more complex paths, which extend the indoor 2D positioning of pedestrian to 3D space. Although more experiments pose more challenges to the algorithm, the positioning result is more convincing.

(2) To provide a baseline for algorithm comparison, detailed experimental settings were given in our study, such as the shape, length, and number of turns for each type of walking path. Furthermore, both absolute and relative positioning errors are calculated, which are quantitative indexes for more accurate evaluation of positioning performance. Some of these are missing in [36,37,38].

(3) In our study, the PDR is performed not only by using the measurements of accelerometer and gyroscope (as done in [35]), but also by fusing the measurements of the magnetometer. In addition, instead of merging the magnetometer data all the time (as done in [17]), a threshold is set to exclude the unusable magnetometer data with severe interference.

Although the experimental results demonstrate that the algorithm implemented in this paper can achieve good 3D positioning results in indoor environments, there is still a lot of work to be done for practical applications. The realization of particle filter-based map-matching algorithm can bring many benefits to indoor pedestrian positioning technology. However, when the pedestrian trajectory deviates too seriously from the ground truth, all propagated particles might fall into the inaccessible region, making the particle filter fail to avoid wall-crossing problem. Generally, the filtered trajectory matches well with the floor plan. However, due to the lack of ground truth in this experiment, the algorithm performance cannot be adequately evaluated.

In the 3D indoor positioning scenario, the pedestrian has to pass through successive floors of the building. The experienced indoor environments is more complex than the outdoor environments, especially the geomagnetic environment. According to the experimental results shown, the heading estimates during level walking is basically at an acceptable level, whereas the heading drift becomes serious during stair climbing. Stair climbing poses a huge challenge to the algorithm in terms of the estimation of heading angle, stride length, and height change. Several reasons might account for this phenomenon, such as (1) the limited accuracy of smartphone’s built-in sensors; (2) the change of magnetic property during floor-to-stair transition; (3) the applicability of gait model when ascending or descending stairs; (4) the frequent minor movements of pocket-worn smartphone.

## 6. Conclusions and Future Work

In this paper, a 3D indoor pedestrian positioning algorithm is proposed based on a pocket-worn smartphone, by fusing the available indoor map content with the sensed walking data from the smartphone’s built-in sensors, including accelerometer, gyroscope, magnetometer, and barometer. Such an algorithm is a self-contained, cost-effective and easy-to-use positioning solution, which can update a pedestrian’s location in any environments at a frequency of about 1 Hz, and more importantly requires no additional peripheral devices except for the ubiquitous smartphone. The 3D positioning process involves the horizontal position calculation by using a PDR algorithm and the vertical height determination by using a pressure-to-height conversion algorithm. Thus, the 3D positioning algorithm consists of four sub-algorithms, i.e., attitude estimation, stride segmentation, stride length calculation and height determination. To prevent the wall-crossing problem of the PDR results, a map-matching algorithm based on a particle filter is implemented. The experimental results highlight the potential application of the 3D positioning algorithm in indoor environments.

In future work, we will consider using an absolute positioning method to obtain the absolute position reference in indoor environments, which can be image-based, Wi-Fi-based or Bluetooth-based. In addition, we will consider implementing pedestrian positioning online or near-online by using the mobile computing resources. Besides, as subjects could carry a phone in different ways, other locations for carrying a smartphone will be considered, such as body, belt, pocket, hand, or bag, to prevent constraining how the smartphone was worn.

## Figures and Tables

**Table 1:** Experimental results of pedestrian dead reckoning for walking along path 1#.

Trial	Average Stride Duration	Stride Cadence	Average Stride Length	Stride Count		Estimated Distance	Estimated Final Position	Final Position Error	
				Detected	Truth			Absolute	Relative
1st	1.06 s	55 stride/min	1.28 m	71	71	90.32 m	(−0.71, −0.23)	0.75 m	0.83%
2nd	1.00 s	59 stride/min	1.32 m	70	70	90.56 m	(−0.46, −0.13)	0.48 m	0.53%
3rd	1.13 s	55 stride/min	1.32 m	69	69	90.74 m	(−0.98, −0.44)	**1.07 m**	**1.19%**
4th	1.01 s	58 stride/min	1.32 m	69	69	90.72 m	(−0.39, 0.32)	0.50 m	0.56%
5th	1.03 s	58 stride/min	1.31 m	70	70	90.54 m	(−0.67, 0.14)	0.68 m	0.76%
Mean	1.05 s	57 stride/min	1.31 m	70	70	90.58 m	--	0.67 m	0.77%

**Table 2:** Experimental results of particle filter-based map matching for walking along path 1#.

Reference Point	Point Location	Estimated Distance	Distance Error (Before)		Distance Error (After)	
			Absolute	Relative	Absolute	Relative
1	(40.5, 25.4)	90.74 m	0.59 m	0.65%	0.11 m	0.12%
2	(40.5, 14.7)	90.74 m	0.15 m	0.16%	0.02 m	0.02%
3	(59.3, 25.4)	90.74 m	1.25 m	1.38%	0.19 m	0.20%
4	(59.3, 14.7)	90.74 m	1.02 m	1.12%	0.73 m	0.80%
5	(68.0, 20.9)	90.74 m	0.30 m	0.33%	0.06 m	0.07%
6	(31.9, 20.9)	90.74 m	0.33 m	0.36%	0.20 m	0.22%
Mean	--	90.74 m	**0.61 m**	**0.67%**	**0.22 m**	**0.24%**

**Table 3:** Experimental results of particle filter-based map matching for walking along path 2#.

Reference Point	Point Location	Estimated Distance	Distance Error (Before)		Distance Error (After)	
			Absolute	Relative	Absolute	Relative
1	(31.9, 20.9)	174.63 m	1.31 m	0.75%	0.26 m	0.15%
2	(68.0, 20.9)	174.63 m	0.7 m	0.40%	0.10 m	0.06%
3	(68.0, 25.4)	174.63 m	1.27 m	0.73%	0.20 m	0.11%
4	(68.0, 14.7)	174.63 m	1.16 m	0.66%	0.52 m	0.30%
5	(50.0, 25.4)	174.63 m	0.47 m	0.27%	0.21 m	0.12%
6	(50.0, 14.7)	174.63 m	0.12 m	0.07%	0.08 m	0.02%
7	(20.0, 20.9)	174.63 m	1.40 m	0.8%	0.12 m	0.07%
8	(7.47, 20.9)	174.63 m	0.57 m	0.33%	0.08 m	0.05%
9	(7.47, 12.0)	174.63 m	0.21 m	0.12%	0.03 m	0.02%
Mean	--	174.63 m	0.80 m	0.46%	0.18 m	0.10%
